# Right Atrium Mass Assessed with 18F-FDG PET/CT Scan Turns Out to Be an Uncommon Relapse of Testicular Diffuse Large B-cell Lymphoma: A Case Report

**DOI:** 10.3390/diagnostics10110987

**Published:** 2020-11-23

**Authors:** Stefano Panareo, Luca Urso, Ivan Santi, Gian Matteo Rigolin, Antonio Cuneo, Corrado Cittanti, Mirco Bartolomei

**Affiliations:** 1 Nuclear Medicine Unit, Oncological Medical and Specialists Department, University Hospital of Ferrara, Via Aldo Moro n.8, 44124 Cona Ferrara, Italy; m.bartolomei@ospfe.it; 2 Department of Translational Medicine and for Romagna, University of Ferrara, 44121 Ferrara, Italy; rsulcu@unife.it (L.U.); ctc@unife.it (C.C.); 3 Nuclear Medicine Unit, Services Department, Hospital of Ascoli Piceno, 63100 Ascoli Piceno, Italy; ivan.santi77@gmail.com; 4 Hematology Section, Department of Medical Sciences, University of Ferrara–University Hospital of Ferrara, 44124 Cona Ferrara, Italy; gianmatteo.rigolin@unife.it (G.M.R.); antonio.cuneo@unife.it (A.C.)

**Keywords:** cardiac neoplasms, testicular tumors, diffuse, large B-Cell, lymphoma, PET CT Scan

## Abstract

We report the case of a 71-year-old man affected by testicular large B-cell lymphoma (DLBCL), treated with right orchiectomy and first-line chemotherapy (R-CHOP, 8 cycles). A complete remission was obtained after therapy. Twenty-two months after the primary diagnosis the patient suddenly presented dyspnoea and superior vena cava syndrome; thus, he underwent a CT scan that revealed a large mass in the right atrium, expanding to the superior vena cava. A differential diagnosis between a neoplastic mass and a clot was proposed. The subsequent MR did not clarify the nature of the mass; therefore, the patient underwent an 18F-FDG PET/CT scan (PET/CT), after a specific preparation to reduce fluoro-deoxyglucose (FDG) myocardial uptake. PET/CT revealed an intense FDG uptake involving the whole mass (SUVmax 9.4), suggestive for neoplasm and confirmed by the subsequent endocardiac biopsy. The patient was treated with 8 cycles of R-COMP, obtaining a complete remission, as indicated by the PET/CT performed after the seventh cycle of therapy. The case that we are reporting highlights that DLBCL can have an uncommon relapse presentation in the atrium. PET/CT, compared to conventional imaging, can be a valuable tool to detect early and better characterize cardiac lesions in order to improve the poor prognosis of these conditions.

## 1. Introduction

The role of 18F-FDG PET/CT (PET/CT) in the assessment of patients with lymphoma is well established and this technique is routinely used, respectively, for staging (baseline study), for the response to treatment and residual disease evaluation, and for the identification of possible relapses. 

Although uncommon, cardiac lymphoma localizations can have a huge impact on a patient’s survival and quality of life and PET/CT can play an important role in their detection.

Conventional imaging, like CT or MR, can provide anatomical details and extension of a potentially heteroplastic cardiac mass, but PET/CT can assess the metabolic activity and, therefore, reveal the aggressive nature of a lesion. 

However, physiological FDG uptake in myocardial tissue is usually variable and unpredictable and can interfere with the study of this region. In order to minimize this phenomenon, the patient should be asked to undergo a preparation consisting of a fat meal the day before the examination and of fasting at least for the twelve hours preceding the scan.

## 2. Case Report

The study was conducted in accordance with the Declaration of Helsinki and national and institutional standards. Written informed consent was obtained from the patient.

In January 2017, a 71-year-old man was diagnosed with testicular diffuse large B-cell lymphoma (DLBCL). He underwent right orchiectomy and was subsequently treated with eight cycles of R-CHOP first-line chemotherapy (February–July 2017). The response-to-treatment evaluation was performed with CT and PET/CT imaging after six cycles of therapy (mid-treatment scan) and a complete early response was proved.

**Figure 1:**
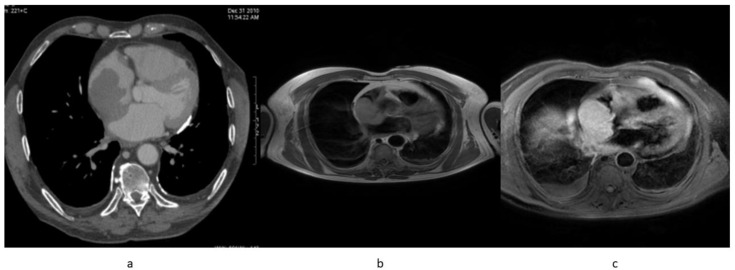
January 2020; (**a**) CT scan showing a right atrial mass hypointens after contast enhanced agent and apparent thickening of the left atrial wall; (**b**) MR with sequences BB T1, confirming the right atrial mass: (**c**) BBT1 delayed gadolinium images sequence showing a lesional delayed enhancement.

The patient’s follow up was then taken in charge by the Haematology Unit. In December 2019, 22 months after the primary diagnosis, the patient suddenly began suffering from dyspnoea with minimal exertion, superior vena cava syndrome, and collar of Stokes: He was admitted to the Emergency Room and afterwards to the Haematology Unit for an adequate treatment. A CT scan performed in January 2020 showed a 60 × 44-mm mass in the right atrium, with a longitudinal extension of 80 mm, partially including superior vena cava, right jugular vein, and left brachiocephalic vein up to the left jugular vein (Figure 1a).

**Figure 2:**
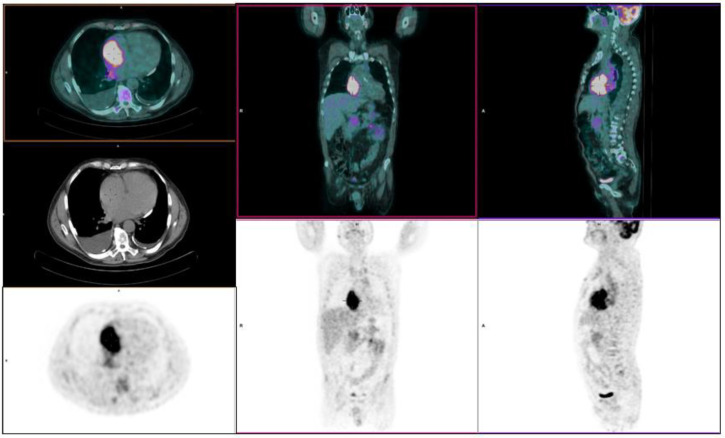
January 2020; 18F-FDG-PET/CT before therapy showing an intense uptake in the right atrium (SUV max 9.4) expression of high metabolic activity. Subsequent biopsy examination confirmed right atrial lymphoma relapse.

He was further studied with cardiac MR in order to clarify the nature of that mass: BB T1 and T2 (also with FAT-SAT sequences) were performed, but the study was not conclusive concerning the differential diagnosis between clot and lymphoma lesion (Figure 1b,c). The subsequent echocardiography only confirmed the already known mass, without adding any hint for the differential diagnosis. Thus, the Haematology Unit decided to perform a PET/CT (January 2020), which revealed a right pleural effusion and, in a context of cardiomegaly, an intense FDG uptake involving the whole right atrium with a SUVmax of 9.4, indicating a gluco-metabolically active lesion rather than a clot, suggestive of neoplasm. No metabolic activity was observed in the upper neck veins (Figure 2). An endocardiac biopsy finally confirmed the clinical suspicion of DLBCL replicative lesion.

**Figure 3:**
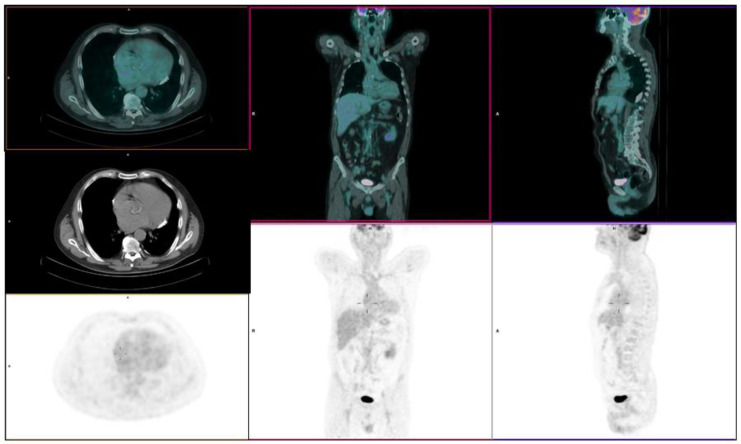
June 2020; 18F-FDG PET/CT after 7 cycles of chemotherapy (R-COMP) showing an early complete response to treatment.

The patient was therefore treated with 8 cycles of chemotherapy (R-COMP), thrombotic prophylaxis, steroids, diuretic, antibiotic therapy, and neutrophil growth factors for neutropenia prophylaxis. A fast resolution of previously reported symptoms was obtained. During therapy, an episode of urinary infection by Klebsiella Pneumoniae, treated with antibiotic, and an episode of atrial fibrillation with spontaneous resolution occurred. The chemotherapy was well tolerated, and the PET/CT performed after the seventh cycle of R-COMP (June 2020) showed a complete disease remission (SUVmax of 1.9 versus previous value of 9.4), associated with a complete resolution of the right pleural effusion previously detected (Figure 3). US examination after treatment completion showed a thickening of the right atrial wall in the absence of the previously detected mass.

## 3. Discussion

Every cardiac mass is potentially lethal, either benign or malignant. Almost 75% of primary cardiac masses are benign, but, when malignant, they are mainly sarcomas or lymphomas [1]. Secondary cardiac involvement is far more common than primary cardiac tumours and it has been documented in 8.7–27.2% of clinical cases of lymphoma [2]. The majority of intracavitary tumors develop on the right heart [3], but the reason of this preferred localization remains unclear [4].

The onset of a secondary cardiac mass typically occurs a few years after primary Non-Hodgkin lymphoma (NHL) diagnosis with a median of 20 months after diagnosis: Our case meets literature findings, having been detected 22 months after diagnosis [5]. Cardiac involvement by lymphoma in autopsy has been described in 16% of patients with Hodgkin disease and 18% of patients with NHL [5]. Even if, sometimes, the myocardial involvement is important, clinical signs and symptoms of cardiac dysfunction (such as chest pain, dyspnoea, arrhythmia, and oedema) may remain undetectable with cardiac involvement being found only after a patient’s death. Patients affected by malignant lymphoma associated with cardiac lesions are at high risk of sudden death, even if they are at an early stage based on clinical findings. Their prognosis is poor and most of these patients die before beginning any therapy [5,6,7,8]. This poor outcome may be, in most cases, due to a delayed diagnosis and treatment. The available literature suggests that systemic chemotherapy is the only effective therapy, with, however, a theoretical risk of cardiac wall perforation as drawback [9,10]. In our case, eight cycles of R-COMP led to a complete remission without significant complications.

Conventional diagnostic techniques show limitations in the early diagnosis and characterization of cardiac involvement from malignant lymphoma. In our case, MRI, cardiac US, and PET provided more specific findings than CT. Echocardiography is the first non-invasive way to examine the four chambers of the heart and pericardium, but the trans thoracic approach is burdened with a restricted acoustic window. Trans esophageal echocardiography (TEE) provides a larger imaging field with higher sensitivity compared to the trans thoracic approach [11].

CT shows morphology, location, and extension of cardiac masses with a larger field of view, while MR signal intensity with contrast enhancement results in better-quality images as far as anatomy, blood flow, and cardiac function are concerned [12,13]. 

PET/CT imaging has been reported to reveal previously unsuspected cardiac involvement and, like in our case, to characterize the extension and the metabolic activity of the cardiac mass and subsequently to assess the response to therapy [14,15]. It is recommended to adopt a proper preparation in order to reduce myocardial FDG uptake and obtain a favorable tumor to background contrast: The patient has to fast for at least twelve hours before the scan and have a fat meal the day before the examination (starting at least 18 h prior to examination).

In our case, it was possible to better characterize the tumor activity and to appreciate its complete response to chemotherapy before echocardiography.

## 4. Conclusions

The case that we are reporting highlights that lymphoma can present in the atrium, and that PET/CT can be a valuable tool to detect early and better characterize lesions in the heart compared to conventional imaging. In fact, the atrial mass was initially thought to be a clot: US, CT, and MR were not able to clarify its nature, whereas PET/CT allowed to suspect a lymphoma relapse later confirmed by biopsy. It is important to diagnose cardiac involvement, which is a clinically underestimated occurrence that determines a poor prognosis: PET/CT turned out to be especially valuable in this case.

There are not many cases of cardiac lymphoma studied with PET/CT in the literature at present. In the future, this imaging technique can have a role in improving conventional imaging performance, adding the metabolic evaluation of lesions, in order to set a tailored therapy and a proper patient management.
